# Self-Crosslinkable Oxidized Alginate-Carboxymethyl Chitosan Hydrogels as an Injectable Cell Carrier for In Vitro Dental Enamel Regeneration

**DOI:** 10.3390/jfb13020071

**Published:** 2022-06-01

**Authors:** Fatemeh Mohabatpour, Zahra Yazdanpanah, Silvana Papagerakis, Xiongbiao Chen, Petros Papagerakis

**Affiliations:** 1 Division of Biomedical Engineering, University of Saskatchewan, 57 Campus Dr., Saskatoon, SK S7N 5A9, Canada; fatemeh.mohabatpour@usask.ca (F.M.); zay413@mail.usask.ca (Z.Y.); 2 College of Dentistry, University of Saskatchewan, 105 Wiggins Rd, Saskatoon, SK S7N 5A9, Canada; 3 Department of Surgery, College of Medicine, University of Saskatchewan, 107 Wiggins Rd, Saskatoon, SK S7N 5A9, Canada; 4 Department of Mechanical Engineering, University of Saskatchewan, 57 Campus Dr., Saskatoon, SK S7N 5A9, Canada

**Keywords:** dental stem cells, enamel regeneration, in situ forming injectable hydrogels, regenerative dentistry

## Abstract

Injectable hydrogels, as carriers, offer great potential to incorporate cells or growth factors for dental tissue regeneration. Notably, the development of injectable hydrogels with appropriate structures and properties has been a challenging task, leaving much to be desired in terms of cytocompatibility, antibacterial and self-healing properties, as well as the ability to support dental stem cell functions. This paper presents our study on the development of a novel self-cross-linkable hydrogel composed of oxidized alginate and carboxymethyl chitosan and its characterization as a cell carrier for dental enamel regeneration in vitro. Oxidized alginate was synthesized with 60% theoretical oxidation degree using periodate oxidation and characterized by Fourier Transform Infrared spectroscopy, proton nuclear magnetic resonance spectroscopy, and Ultraviolet-visible absorption spectroscopy. Then, hydrogels were prepared at three varying weight ratios of oxidized alginate to carboxymethyl chitosan (4:1, 3:1, and 2:1) through Schiff base reactions, which was confirmed by Fourier Transform Infrared spectroscopy. The hydrogels were characterized in terms of gelation time, swelling ratio, structure, injectability, self-healing, antibacterial properties, and in vitro characterization for enamel regeneration. The results demonstrated that, among the three hydrogels examined, the one with the highest ratio of oxidized alginate (i.e., 4:1) had the fastest gelation time and the lowest swelling ability, and that all hydrogels were formed with highly porous structures and were able to be injected through a 20-gauge needle without clogging. The injected hydrogels could be rapidly reformed with the self-healing property. The hydrogels also showed antibacterial properties against two cariogenic bacteria: *Streptococcus mutans* and *Streptococcus sobrinus*. For in vitro enamel regeneration, a dental epithelial cell line, HAT-7, was examined, demonstrating a high cell viability in the hydrogels during injection. Furthermore, HAT-7 cells encapsulated in the hydrogels showed alkaline phosphatase production and mineral deposition, as well as maintaining their round morphology, after 14 days of in vitro culture. Taken together, this study has provided evidence that the oxidized alginate-carboxymethyl chitosan hydrogels could be used as an injectable cell carrier for dental enamel tissue engineering applications.

## 1. Introduction

Dental caries represent the most common oral disease caused by microbial biofilms, which results in enamel damage and demineralization, cavity formation, and tooth destruction [1,2]. The loss of enamel forming cells, ameloblasts, after tooth eruption causes enamel to have no capacity to self-regenerate [3]. Thus, developing innovative approaches to regenerate dental enamel are needed to tackle the loss of dental enamel tissue and to address its constant challenge of maintaining its integrity within the oral environment [4]. Tissue engineering has shown a remarkable promise as a therapeutic option in restorative dentistry to regenerate damaged or lost dental tissues [5,6]. Tooth tissue engineering employs three main elements including dental stem cells, scaffolds, and growth factors to reconstruct new dental tissues that resemble the structure and function of the native tissue [5,7,8]. Dental enamel is developed through the amelogenesis process during which enamel crystals are grown in a gel-like organic matrix secreted by ameloblasts [9]. Hydrogels have been used as models for enamel mineralization due to their capacity to mimic the in vivo enamel matrix, and have shown to promote the regrowth of enamel crystals and to form enamel-like structures [10,11].

Injectable hydrogels have shown a great potential as cell carriers and/or biomolecule-delivery systems in regenerative medicine and cancer treatment [12,13,14,15]. Injectable hydrogels can be formed in situ with many advantages over the pre-formed hydrogels that are shaped prior to implantation [16,17,18]. These advantages include the minimally invasive procedure needed for applying injectable hydrogels to the defected site (thus leading to reduced risk of infection and decreased patient discomfort), the capacity to fill defects with irregular shapes, and the easy incorporation of cells and biomolecules into the hydrogel solution prior to injection [16,17,18]. Once injected, hydrogels need to be appropriately crosslinked or gelled for their functions. Physical crosslinking, such as ionic or thermal gelation, occurs in milder conditions, but the crosslinked hydrogels typically have poor mechanical properties [19]. In contract, hydrogels formed via chemical crosslinking have shown to have stronger mechanical properties and a more stable structure [19,20,21]. The Schiff base reaction is of a chemical crosslinking mechanism that occurs between aldehyde and amino groups of polymers [22]. As this reaction does not involve the use of chemical crosslinker(s) or other external stimulations, it is considered as a cell-friendly mechanism particularly suitable for cell incorporation [23,24]. Furthermore, the dynamic Schiff base crosslinking confers self-healing properties to the hydrogels, allowing them to spontaneously recover following damage as well as to provide cells with a more protective environment [25,26]. Hydrogels have been developed from the Schiff base reaction [26,27] and among them, the hydrogel formed from the oxidized alginate and carboxymethyl chitosan (CMC) stands out for their distinct, yet complementary, properties including injectability, self-healing, and antibacterial properties [23,28,29]. Alginate is a natural anionic polysaccharide that has been widely used in dental impression and tissue engineering owing to its appealing biocompatibility and its ability to be gelled under mild conditions in the presence of divalent cations [12,15,30,31]. However, alginate has low cell attachment properties and a low degradation rate [32]. Alginate can be modified through an oxidation reaction using periodate (an anion composed of iodine and oxygen) that converts the hydroxyl groups in the backbone of alginate to aldehyde groups [12,15,33]. Oxidized alginate has reported to have an improved biocompatibility and degradation rate in aqueous environments [28,32]. CMC is a water-soluble derivative of chitosan that has antibacterial activity, appropriate biocompatibility, and cell interaction properties and as such, chitosan has been widely used in various tissue regeneration applications [34,35,36,37,38]. It has been illustrated that injectable hydrogels with a self-healing ability have been promising in many applications such drug/cell delivery [26,39,40,41,42] and wound healing [43,44]. To the best of our knowledge, the so-formed oxidized alginate-CMC hydrogel has not yet been investigated for the regeneration of dental tissues.

The aim of this study was to develop such an injectable oxidized alginate-CMC hydrogel and employ it as a cell carrier for dental enamel caries healing and tissue regeneration. Oxidized alginate was synthesized using sodium meta-periodate, followed by chemical characterization and the determination of the degree of oxidation. Then, the hydrogels were prepared from oxidized alginate and CMC at three different weight ratios. The properties of hydrogels were evaluated in terms of gelation time, swelling ratio, chemical and morphological characterizations, as well as their injectability, self-healing, and antibacterial properties. The suitability of self-crosslinkable hydrogels for in vitro enamel regeneration was evaluated by the encapsulation and in vitro culture of dental epithelial cell line, HAT-7, followed by an assessment of cell viability, cell morphology, and mineral formation in the cell-laden hydrogels.

## 2. Materials and Methods

### 2.1. Synthesis of Oxidized Alginate

Oxidized alginate was synthesized according to previously reported protocols [45,46]. Two grams of sodium alginate powder (Sigma-Aldrich, St. Louis, MO, USA) was dispersed in 10 mL ethanol. Then, 1.28 g of sodium meta-periodate (Thermo Fisher Scientific, Waltham, MA, USA) was dissolved in 10 mL water (the molar ratio of sodium meta-periodate to repeating units of alginate was 0.6 to produce oxidized alginate with 60% theoretical degree of oxidation) and added to the alginate dispersion drop by drop and continuously stirred at room temperature in the dark for 6 h. The reaction was stopped by adding 2 mL ethylene glycol (Sigma-Aldrich) and stirred for an additional 30 min. The mixture was then dialyzed against water using Seamless Cellulose Dialysis Tubing (molecular weight cut-off 12 kDa, Fisher Scientific) with several changes of water. The oxidized alginate solution was freeze-dried for 3 days and kept at 4 °C until used. To characterize the composition of oxidized alginate and unmodified alginate, Fourier Transform Infrared (FTIR) spectroscopy was performed by using an IlluminatIR II inVia Reflex (Smiths Detection, Danbury, CT, USA) equipped with an attenuated total reflectance (ATR) objective. The spectra were drawn as transmittance to wavenumber. Transmittance represents the areas where certain molecular bonds in the polymer absorb the light passing through the samples [47]. The successful synthesis of oxidized alginate was confirmed by proton nuclear magnetic resonance (^1^HNMR) spectroscopy using a Bruker Advance 500 MHz spectrometer (Bruker, Billerica, MA, USA). Oxidized alginate and alginate were dissolved in deuterium oxide and were transferred into NMR tubes and the spectra were taken at room temperature.

### 2.2. Determination of the Oxidation Degree

The oxidation degree of synthesized oxidized alginate was determined by ultraviolet-visible (UV-Vis) absorption spectroscopy [48]. The 20% (*w*/*v*) potassium iodide (Fisher Scientific) and 1% (*w*/*v*) starch solutions (Fisher Scientific) in buffer phosphate (pH 7) were mixed in 1:1 volume ratio to prepare the indicator solution. One mL of oxidation reaction mixture prior to adding ethylene glycol was diluted with 250 mL distilled water. Three mL of diluted solution was added to 1.5 mL of indicator solution and then 0.5 mL of distilled water was added to the solution. The absorbance of the diluted solution was measured at 486 nm to determine the concentration of the sodium meta-periodate in the sample using the standard curve created for sodium meta-periodate solutions. The consumed amount of the periodate was calculated by subtracting the amount of sodium meta-periodate before and after oxidation reaction. The oxidation degree (OD) of alginate was calculated using the following equation [49]:

$$\mathrm{OD}\;(\%)=\frac{{\;m}_{w}\times \;n\;}{m}\times 100$$

where m_w_ is the molecular weight of the repeating units of sodium alginate that is equal to 198 g/mol; m is the initial amount of sodium alginate (g); and n is the amount of sodium meta-periodate (mol). To calculate the theoretical oxidation degree, the initial amount of sodium meta-periodate (mol) is considered as n, while for the actual oxidation degree n is considered as the consumed amount of sodium (mol).

### 2.3. Self-Crosslinkable Hydrogel Formation 

Three different concentrations of oxidized alginate (10%, 15%, and 20% (*w*/*v*)) and 7.5% (*w*/*v*) CMC (Santacruz Biotechnology, Dallas, TX, USA) solutions were separately prepared in phosphate buffered saline (PBS) under stirring at room temperature. Then, each concentration of oxidized alginate solutions was gently mixed with carboxymethyl chitosan solution at a fixed volume ratio of 60:40 (*v*/*v*%) to obtain hydrogel solutions with three different weight ratios of 4:1, 3:1, and 2:1 (Table 1). Hydrogel solutions were then placed in 37 °C to allow for the gelation through self-crosslinking to occur.

### 2.4. Gelation Time

Gelation time of self-crosslinked oxidized alginate/carboxymethyl chitosan was determined by the tube inversion method [26]. Briefly, hydrogel solutions with the compositions described above were prepared in 15 mL glass vials and placed in 37 °C. Gelation of hydrogels were monitored by inverting the tube at 37 °C and the gelation time was recorded when hydrogel solutions stopped flowing.

### 2.5. Chemical Characterization of Self-Crosslinked Hydrogels

To evaluate the crosslinking between oxidized alginate and carboxymethyl chitosan, FTIR spectroscopy was performed using the hydrogel sample with highest concentration of oxidized alginate (with 4:1 ratio of OAlg:CMC). Briefly, hydrogel samples were prepared as described before and were crosslinked at 37 °C according to the gelation time measured by tube inversion test and then were instantly placed in −80 °C and lyophilized for 24 h. The spectra of lyophilized hydrogels were recorded using the same instrument as previously mentioned and were compared with oxidized alginate and carboxymethyl chitosan.

### 2.6. Swelling Test

Crosslinked hydrogels were immersed in PBS solution and allowed to completely swell. After incubating and equilibrating the samples at 37 °C for 24 h, hydrogels were removed, swollen weights (W_s_) were measured, samples were dried under vacuum, and dry weights were measured (W_d_). Swelling ratio was calculated according to the swelling ratio = (W_s_ − W_d_)/W_d_ equation.

### 2.7. Microstructural Observation of the Self-Crosslinkable Hydrogels

The hydrogels’ morphology was analyzed by scanning electron microscopy (SEM). The self-crosslinked hydrogels were lyophilized at −50 °C and placed on the aluminum stubs and sputter coated with a thin layer of gold using a sputter coater (Q150T ES, Quorum Technologies, Laughton, East Sussex, UK). Samples were imaged under vacuum using a field scanning electron microscope (Hitachi High-Tech SU8010, Chiyoda, Tokyo, Japan).

### 2.8. Injectability and Self-Healing Assays

The injectability of the hydrogels was assessed using a previously reported method [50,51]. The hydrogel with a 2:1 ratio of OAlg:CMC was chosen as an example. OAlg and CMC solutions were added to a 5 mL glass vial, vortexed until a homogenous mixture was obtained, and then allowed for the sol–gel transition to occur. After gelation, the disk-shaped hydrogel was loaded into a 3 mL syringe and injected using a 20-gauge needle to write four letters “ABCD” on a glass microscope slide. In addition, the hydrogels were injected into a 10 mL beaker that was kept at 37 °C for 10 min to allow for the self-healing of the hydrogels. Photos of one single piece of hydrogel were taken to record the self-healing property. The stability of the self-healed hydrogels was assessed by immersion in PBS.

### 2.9. Antibacterial Properties 

To assess the antibacterial properties of the OAlg-CMC hydrogels, the inhibition zone test [52] was performed using two cariogenic bacteria commonly found in the dental plaque biofilm, *Streptococcus mutans* and *Streptococcus sobrinus.* Both bacterial strains were incubated in Bacto^TM^ Brain Heart Infusion broth (BHI, NJ, Becton, Dickinson and Company, (BD), USA) at 37 °C overnight to obtain the bacterial suspensions. Then, 200 μL of bacterial suspensions (*Streptococcus mutans*, 10^6^ CFU/mL and *Streptococcus sobrinus,* 10^9^ CFU/mL) were uniformly spread on the surface of agar plates (BBL^TM^ Brain Heart Infusion Agar, Sparks, MD, USA), followed by placing 200 μL of hydrogel samples at all three ratios of OAlg:CMC. After incubation for 24 h at 37 °C, the inhibition zones were measured using image J software (version 2.3.0/1.53f, NIH, Bethesda, MD, USA).

### 2.10. In Vitro Evaluations of Self-Crosslinkable Hydrogels

#### 2.10.1. Cell Culture

HAT-7 dental epithelial cell line originating from the cervical loop epithelium of a rat incisor, (CVCL_RW36) was kindly provided by Dr. Hidemitsu Harada (Iwate Medical University, Iwate, Japan) [53] and were cultured in DMEM/F12 (1:1), HEPES medium (Gibco^®^, Invitrogen, Carlsbad, CA, USA) supplemented with 10% fetal bovine serum (FBS, Gibco^®^, Invitrogen, Carlsbad, CA, USA) and 1% antibiotic (Penicillin-Streptomycin 100X solution, 10,000 units/mL Penicillin/10,1000 μg/mL Streptomycin, Hyclone^TM^, Logan, UT, USA) in a humidified incubator. Medium was changed every 2–3 days.

#### 2.10.2. Cell Encapsulation in Self-Crosslinkable Hydrogels

Oxidized alginate and carboxymethyl chitosan powder were sterilized by exposure to UV light for 90 min. Then, oxidized alginate (10%, 15%, and 20% (*w*/*v*)) and 7.5% (*w*/*v*) carboxymethyl chitosan solutions were separately prepared in PBS under stirring under sterile conditions. HAT-7 cells were sub-cultured twice, counted, and then resuspended in 7.5% carboxymethyl chitosan solutions. Cell-containing CMC solutions were mixed with oxidized alginate solutions at a volume ratio of 40:60 (*v*/*v*%) to obtain hydrogel solutions with weight ratios of 4:1, 3:1, and 2:1, respectively. A final cell density of 400,000 cells/mL was used for live/dead assay and 6 × 10^6^ cells/mL for alkaline phosphatase and alizarin red S staining and SEM analysis. Hydrogel solutions were inserted in the wells of 96-well cell culture plates and kept at 37 °C to be crosslinked through Schiff base reaction. Next, culture media supplemented with 10% FBS and 1% antibiotics was added to the cell-laden hydrogels and kept in the incubator at 37 ℃  and 5% CO_2_. Culture media was changed every 2–3 days.

#### 2.10.3. Cell Viability Assessment

To investigate the effect of hydrogel composition and extrusion pressure during injection on cell viability, a live/dead assay was performed using the LIVE/DEAD Cell imaging kit (Invitrogen, Thermo Fisher Scientific, Carlsbad, CA, USA) according to the manufacturer’s instructions at days 0, 1, and 3 after cell encapsulation. Briefly, 50 microliters of the hydrogel encapsulating HAT-7 cells were either injected to the wells of the 96-well cell culture plates using 20-gauge needles or mixed in the wells of 96-well plates with pipette tips. After 2, 24, and 72 h of culture at 37 °C, cell-laden hydrogels were washed with PBS and stained with the mixed green/red dye. After 15 min incubation at room temperature, constructs were imaged using fluorescent microscope (EVOS M5000 Cell Imaging System, Thermo Fisher Scientific). The number of live (green) and dead (red) cells were quantified by using image J software and viability was calculated with the following equation:

$$\mathrm{Cell}\;\mathrm{viability}=\frac{\left(\mathrm{number}\;\mathrm{of}\;\mathrm{live}\;\mathrm{cells}\right)}{\left(\mathrm{number}\;\mathrm{of}\;\mathrm{live}\;\mathrm{cells}\;+\;\mathrm{number}\;\mathrm{of}\;\mathrm{dead}\;\mathrm{cells}\right)}$$

#### 2.10.4. Alkaline Phosphatase Staining

HAT-7 cells were cultured within oxidized alginate-carboxymethyl chitosan hydrogels for 14 days and then stained with an alkaline phosphatase (ALP) staining kit to detect the expression of alkaline phosphatase, a marker of ameloblast differentiation. Cell-laden constructs were rinsed with PBS twice and cells encapsulated in the hydrogels were fixed and permeabilized using BD Cytofix/cytoperm^TM^ fixation/permeabilization kit (BD Biosciences, San Jose, CA, USA) by incubating the constructs in the fixation and permeabilization solution for 30 min at 4 ℃. Cell-encapsulated hydrogels were washed with 1 × BD Perm/Wash^TM^ buffer and then with PBS, twice each. Then, alkaline phosphatase expression in cell-laden constructs was detected using SIGMAFAST^TM^ BCIP^®^/NBT detection kit (Sigma-Aldrich). The ALP staining reagent was prepared by dissolving 1 tablet from SIGMAFAST^TM^ BCIP^®^/NBT detection kit (Sigma-Aldrich) in 10 mL of distilled water as instructed by the manufacturer. Cell-laden constructs were incubated in ALP staining reagent at 37 °C under dark condition for 24 h. Cell-encapsulated hydrogels were then visualized under a light microscope (EVOS M5000 Cell Imaging System, Thermo Fisher Scientific).

#### 2.10.5. Alizarin Red S Staining

After 14 days of culture, HAT-7-encapsulated oxidized alginate-carboxymethyl chitosan hydrogels were stained with alizarin red S to assess mineralization and calcium deposition. Cells within the hydrogels were fixed and permeabilized as previously described. Then, 2% Alizarin red S (pH 4.2, Santa Cruz Biotechnology, Dallas, TX, USA) was added to cell-laden constructs and incubated for 5 min at room temperature. Cell-laden hydrogels were then rinsed with distilled water twice and imaged using the same light microscope as mentioned above.

#### 2.10.6. Observation of Cell Morphologies in the Self-Crosslinked Hydrogels

After 14 days of culture, the morphology of cells and the mineral deposition in the self-crosslinked hydrogels were examined by scanning electron microscopy (SEM). Cell encapsulated hydrogels with all three weight ratios of oxidized alginate to carboxymethyl chitosan were rinsed with PBS twice and fixed with 2.5% *v*/*v* glutaraldehyde in PBS for 2 h. After washing with PBS and distilled water, three times each, samples were kept at −80 °C and then freeze-dried. The lyophilized call-laden hydrogels were sputter coated with a chromium film with a sputter coater and imaged using the same scanning electron microscope as previously mentioned.

### 2.11. Statistical Analysis

All data are presented as mean ± standard deviation and were statistically analyzed using one-way ANOVA and the post-Tukey test by GraphPad Prism 5 (GraphPad Software, San Diego, CA, USA). A *p* value < 0.05 was considered statistically significant.

## 3. Results

### 3.1. Comparative Chemical Characterization of Synthesized Oxidized Alginate and Alginate Confirmed the Modification of Alginate with Aldehyde Groups

**Figure 1:**
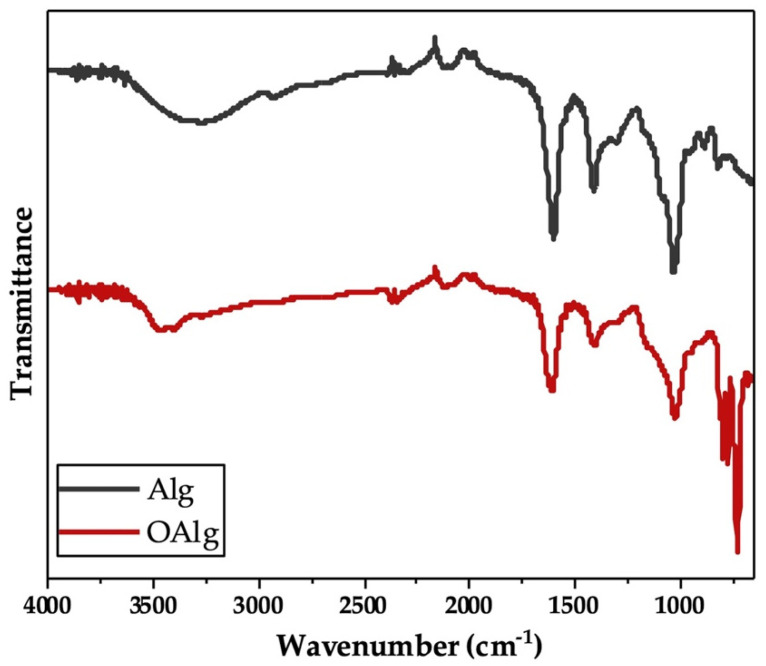
Fourier Transform Infrared-Attenuated Total Reflectance (FTIR-ATR) of alginate (Alg) and synthesized oxidized alginate (OAlg) confirmed the modification of alginate by the addition of aldehyde groups using sodium meta-periodate oxidation reaction that enables oxidized alginate to react with amino containing polymers and form self-crosslinkable hydrogels through Schiff base reaction.

Sodium alginate was synthesized through the oxidation reaction using sodium meta-periodate. During this reaction, hydroxyl groups on carbons two and three (C2 and C3) from the monomeric units of unmodified alginate are oxidized, which leads to the cleavage of the C2–C3 bond and the formation of aldehyde groups in each oxidized repeating units as previously reported [48]. In our study, we characterized the synthesized oxidized alginate using FTIR and ^1^HNMR. FTIR spectra of unmodified alginate and oxidized alginate (Figure 1) exhibited the characteristic peaks at 1605 and 1405 cm^−1^, which are attributed to asymmetric and symmetric stretching modes of carboxyl groups, respectively, as previously reported in other studies [54]. The broad peak that appeared at 3267 cm^−1^ in the spectrum of alginate more likely corresponded to the stretching vibration of hydroxyl groups (-OH), which are known to become weaker and narrower in the spectrum of oxidized alginate [46]. The peaks noted at 815 and 1078 cm^−1^ in the spectrum of alginate are related to the symmetrical C-O-C stretching according to other studies, that are known to be reduced in the spectrum of oxidized alginate [54]. In addition, the new peak that is known to be related to the symmetric vibration of aldehyde groups according to previous studies was not observed at 1735 cm^−1^ in the FTIR spectrum of oxidized alginate [54].

**Figure 2:**
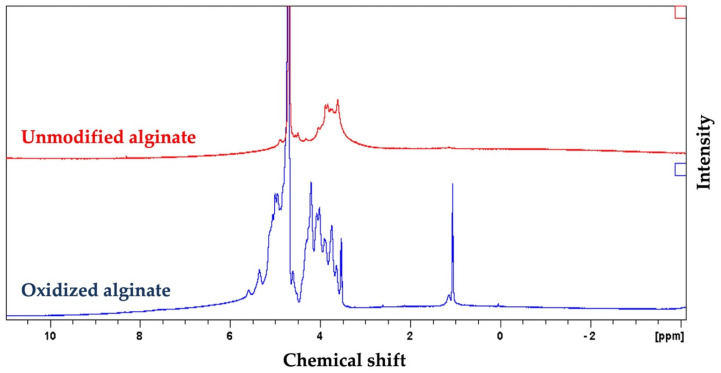
Proton nuclear magnetic resonance spectroscopy (^1^HNMR) spectrum of alginate and synthesized oxidized alginate further confirmed the successful synthesis of oxidized alginate. The appearance of new peaks at 4.2, 5.3, and 5.6 in the spectrum of the oxidized alginate that are absents in the spectrum of alginate are attributed to the oxidation of alginate.

The ^1^HNMR spectrum of oxidized alginate showed two new signals at 5.3 and 5.6 ppm (Figure 2), which are usually assigned to the hemiacetalic protons, according to other studies, confirming the successful synthesis of oxidized alginate [55]. The new signal at 4.2 ppm in the spectrum of oxidized alginate is attributed to the proton of the oxidized guluronic acid unit in the backbone of the polymer according to previous studies [55].

### 3.2. The Experimental Oxidation Degree of Alginate as a Representation of Available Aldehyde Groups for Schiff Based Reaction Was Found to Be Close to the Theoretical Oxidation Degree

After the oxidation reaction, the oxidation degree was determined to be 59.33% by measuring the quantity of consumed sodium meta-periodate using UV-vis absorption spectroscopy, which is very close to the theoretical oxidation degree (60%) and the range reported by other studies [32,48].

### 3.3. Comparative Chemical Characterization of Hydrogel and Alginate and Carboxymethyl Chitosan Confirmed the Formation Self-Crosslinkable Hydrogel through Schiff Base Reaction

**Figure 3:**
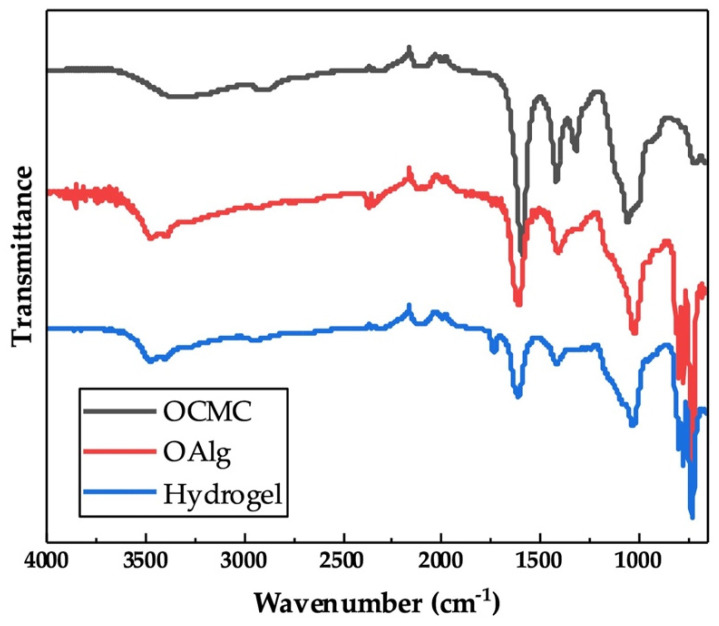
Chemical characterization of self-crosslinked oxidized alginate-carboxymethyl chitosan hydrogel with the weight ratio of 4:1 using Fourier Transform Infrared-Attenuated Total Reflectance (FTIR-ATR) showed the imine bond formation as a result of Schiff base reactions. The appearance of a new peak at 1621 cm^−1^ in the spectrum of hydrogel is attributed to the C=N bonds that are absent in the spectra of oxidized alginate (OAlg) and carboxymethyl chitosan (OCMC).

The chemical crosslinking of hydrogels through the Schiff base reaction assessed by FTIR indicated the appearance of a new peak at 1621 cm^−1^ in the spectrum of OAlg-CMC hydrogels, which is related to Schiff base C=N bonds [56] that were merged with the C=O stretching vibrations of carboxylate in carboxymethyl chitosan observed at 1586 cm^−1^ (Figure 3) [57]. In addition, the peak at 1729 cm^−1^ in the spectrum of hydrogel in Figure 3 is known to be related to the free aldehyde groups in oxidized alginate, according to other studies [54], that were expected to appear in the FTIR spectrum of oxidized alginate that is shown in Figure 1.

### 3.4. Hydrogels with Higher the OAlg:CMC Ratio Showed Faster Gelation Time

**Figure 4:**
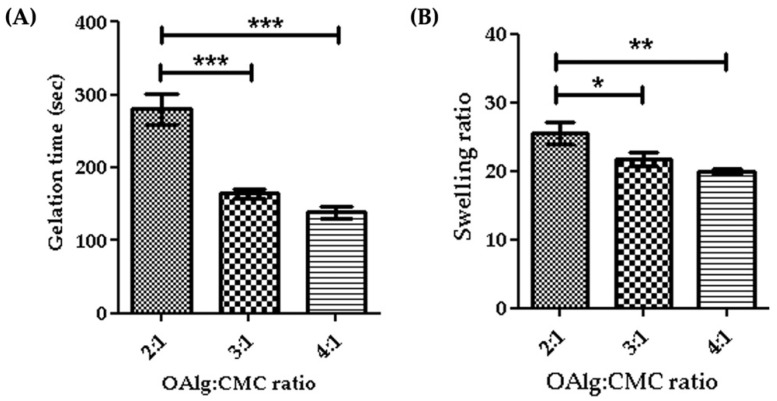
Gelation time (**A**) and swelling ratios (**B**) of self-crosslinked oxidized alginate (OAlg)-carboxymethyl chitosan (CMC) hydrogels with different weight ratios showed that the higher weight ratios of oxidized alginate to carboxymethyl chitosan decreased both the gelation time and the swelling ratio of the hydrogels. Data are shown as mean and error bars represent the standard deviation of three independent experimental replicates (* statistical significance is indicated as *** *p* < 0.001, ** *p* < 0.01, * *p* < 0.05).

Gelation time is considered as the required time for hydrogels to form and complete crosslinking [58]. In this study we assessed the gelation time of self-crosslinked hydrogels for various weight ratios of oxidized alginate and carboxymethyl chitosan and found that the higher weight ratio of oxidized alginate in the hydrogel led to a shorter gelation time (Figure 4A). The gelation time decreased from 4.7 min for the 2:1 ratio OAlg:CMC to 2.3 min for the 4:1 ratio of OAlg:CMC, which could be due to the higher concentration of aldehyde groups for the Schiff base reaction that accelerates the crosslinking reaction and gel formation. This observation is in agreement with previous studies reporting that hydrogels with a quick gelation time are crucial for the clinical application of injectable hydrogels because they can facilitate filling the defective region [58].

### 3.5. Hydrogels with Higher the OAlg:CMC Ratio Showed Lower Swelling Ability 

The quantity of water absorbed into the self-crosslinked hydrogels was determined by a swelling test after a 24 h incubation in PBS. Our results indicated that the swelling ratio significantly decreased by increasing the weight ratio of oxidized alginate and carboxymethyl chitosan (Figure 4B). The swelling ratio of hydrogels is contingent upon crosslinking density and as such hydrogels with a higher crosslinking density have a lower swelling ratio [59]. A higher weight ratio of oxidized alginate provides more aldehyde groups available to react with amino groups for the Schiff base reaction that leads to the formation of more imine bonds and an increased crosslinking density, which in turn lowers the swelling ratio [59].

### 3.6. Morphological Characterization of Self-Crosslinked Hydrogels

**Figure 5:**
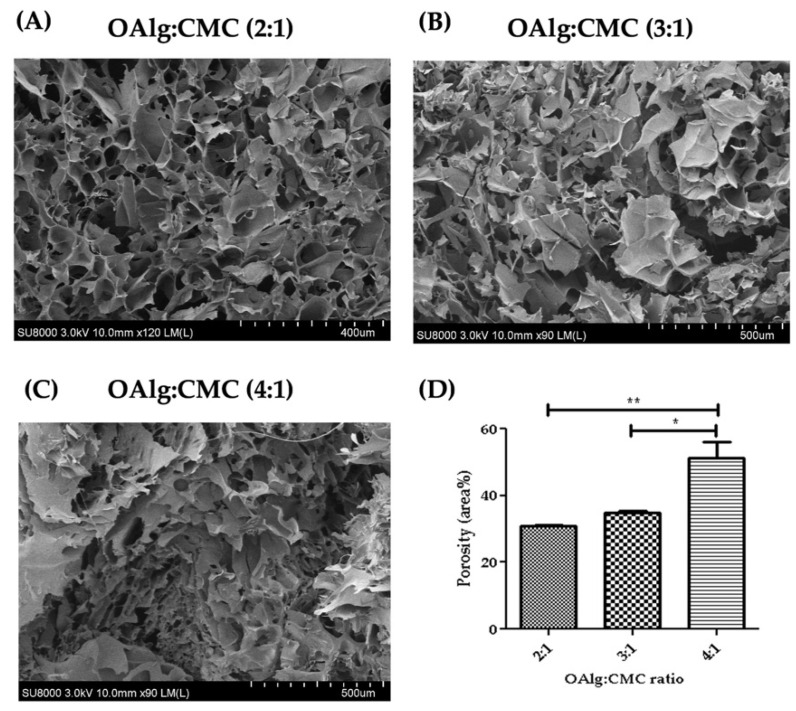
Scanning electron microscopy (SEM) images of self-crosslinked oxidized alginate-carboxymethyl chitosan hydrogels at three different weight ratios (4:1, 3:1 and 2:1) after lyophilization showed the highly porous structure of the hydrogels. The hydrogels with higher weight ratio of OAlg showed smaller pore sizes, higher porosity, and a less homogenous distribution of pore size. Data are shown as mean and error bars represent the standard deviation of two independent experimental replicates (* statistical significance is indicated as ** *p* < 0.01, * *p* < 0.05).

Cross-sectional morphology of the hydrogels observed under SEM showed interconnected and porous structures with pores size of microns (Figure 5). The hydrogels with higher ratio of OAlg showed smaller pore sizes and a less homogenous distribution of pore size. The quantified porosity results shown in Figure 5D revealed significantly higher porosity of the hydrogels with the OAlg:CMC ratio of 4:1 (51.40 ± 4.59) compared to 3:1 (34.72 ± 0.72, *p* < 0.05), and 2:1 (30.88 ± 0.2072, *p* < 0.01) ratios.

### 3.7. Injectability and Self-Healing Properties

**Figure 6:**
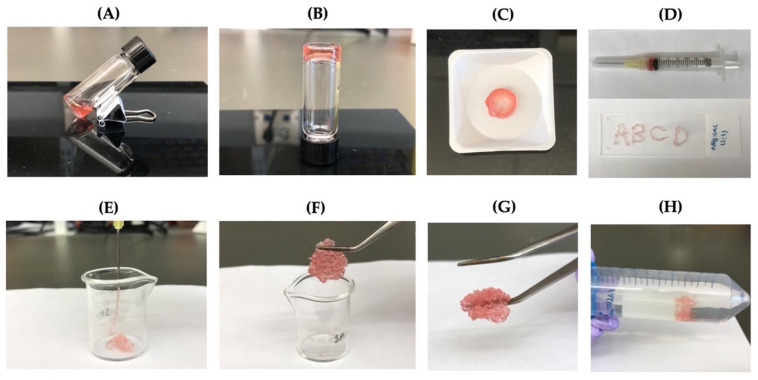
The injectability and self-healing assessments of the self-crosslinkable hydrogels at weight ratios of 2:1 oxidized alginate to carboxymethyl chitosan. (**A**,**B**) Hydrogel sol–gel transition. (**C**,**D**) Hydrogels’ injectability confirmed by drawing of letters “ABCD” using the formed disk-shaped hydrogel as ink after placing it in a 3 mL syringe. (**E**) Hydrogels’ injectability confirmed by extrusion without clogging through a 20-gauge needle into a 10 mL beaker. (**F**,**G**) Self-healing of injected hydrogel fragments and self-healing/reformation in a single piece after 10 min incubation at 37 °C. (**H**) Confirmation of hydrogels stability and integrity after self-healing through immersion in PBS.

The ability of the self-crosslinkable hydrogels for potential applications as injectable cell delivery systems, hydrogels, after gelation were tested by extrusion through a 20-gauge needle (Figure 6A–C). Four letters “ABCD” were successfully drawn using the hydrogels as illustrated in Figure 6D. In addition, OAlg-CMC hydrogels were able to be injected without clogging through the 20-gauge needle into a 10 mL glass beaker (Figure 6E). After 10 min, pieces of the injected hydrogels were rapidly recovered through re-crosslinking, which were successfully reconstituted in a single piece hydrogel (Figure 6F,G). In addition, the self-healed hydrogels maintained their structure when immersed in PBS (Figure 6H).

### 3.8. Assessment of Antibacterial Properties

**Figure 7:**
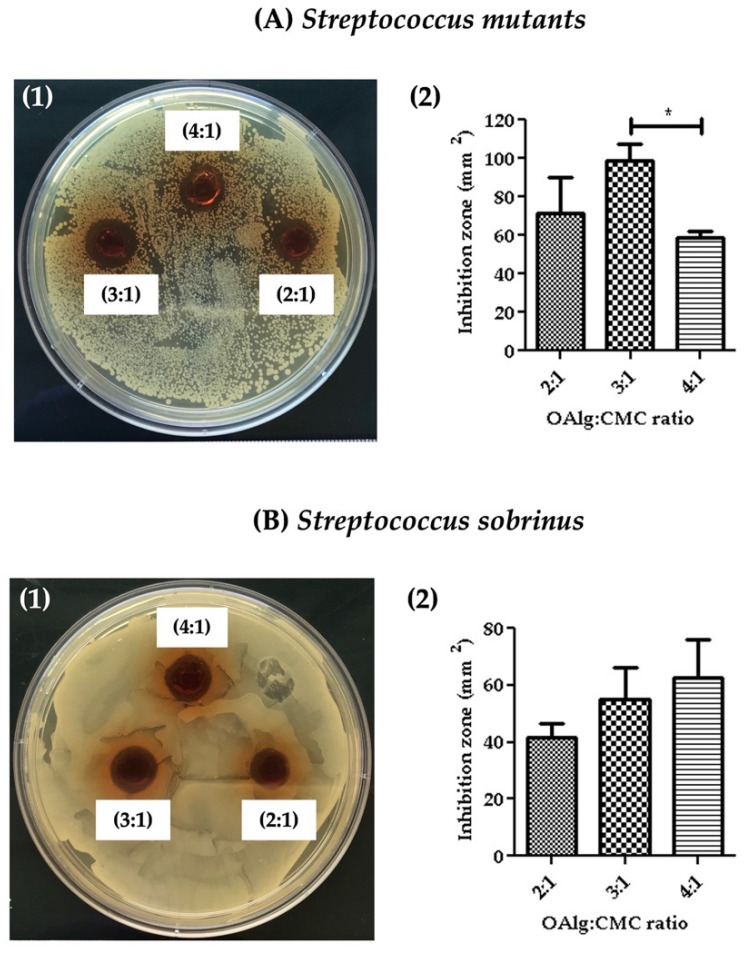
The assessment of the antibacterial activity of self-crosslinked hydrogels at three different weight ratios of oxidized alginate to carboxymethyl chitosan by measuring the hydrogels’ inhibition of bacterial growth against (**A**) *Streptococcus mutans* and (**B**) *Streptococcus sobrinus.* Data are shown as mean and error bars represent the standard deviation of three independent experimental replicates (statistically significant results indicated in the graphs, * *p* < 0.05).

Antibacterial properties of the hydrogels were assessed against *Streptococcus mutans and Streptococcus sobrinus* by measuring the inhibition zone where no bacterial growth was observed. The inhibition zones were quantified using image J software. Hydrogels at all three OAlg:CMC weight ratios showed the growth inhibition against both *Streptococcus mutans* and *Streptococcus sobrinus* (Figure 7). Hydrogels with the weight ratio of 3:1 OAlg:CMC exhibited the larger inhibition zone compared to the other two weight ratios of 4:1 and 2:1; however, only the difference between 3:1 vs. 4:1 ratio was statistically significant with a *p* value < 0.05). These findings suggested stronger antibacterial properties of this group towards *Streptococcus mutans* (Figure 7(A1,A2)). In contrast, although a trend of increased inhibition zone with the increase in the weight ratio was observed against *Streptococcus sobrinus*, the noted differences between the three different groups of hydrogels were not statistically significant (Figure 7B). *Streptococcus mutans* is known to be more frequent in carious lesions compared to *Streptococcus sobrinus* [60].

### 3.9. Live/Dead Assay

**Figure 8:**
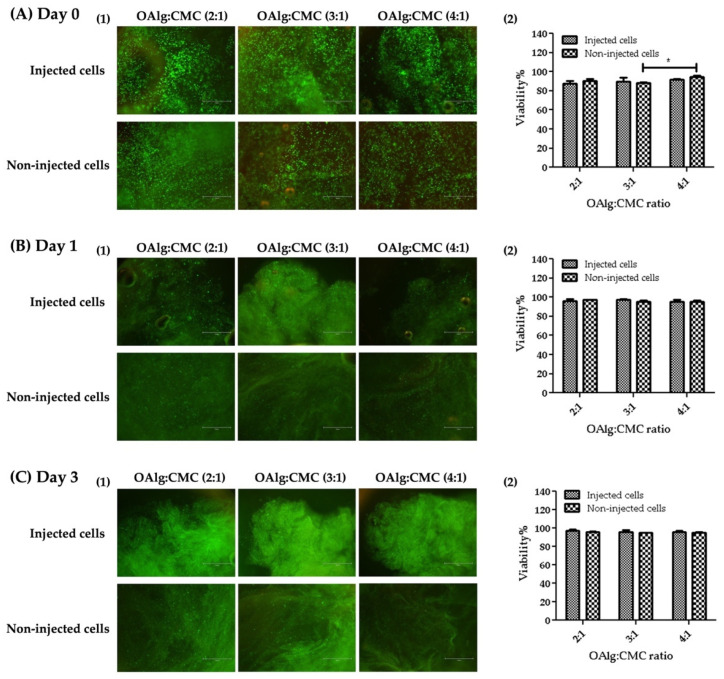
Live/dead staining of HAT-7 cells encapsulated in self-crosslinked oxidized alginate-carboxymethyl chitosan hydrogels at different time points (Days 0, 1, and 3) showed no significant difference in the viability of the injected cells compared to non-injected cells. Data are shown as mean and error bars represent the standard deviation of three independent experimental replicates (* statistically significant results indicated in the graphs, * *p* < 0.05).

The viability of HAT-7 cells in OAlg-CMC hydrogels was assessed using live/dead assays at 0, 1, and 3 days after cell encapsulation or injection. HAT-7 cells loaded-hydrogels were injected through a 20-gauge needle in a 96-well plate. The extruded hydrogel pieces were self-healed and became one piece of hydrogel (Figure 8). Both non-injected and injected HAT-7 cells showed a very high cell viability (over 80%), indicating the cytocompatibility of the self-crosslinked hydrogels in the absence of negative effects of extrusion pressure (during hydrogels injection) on cell survival at all three time points.

### 3.10. Mineralization Assays

**Figure 9:**
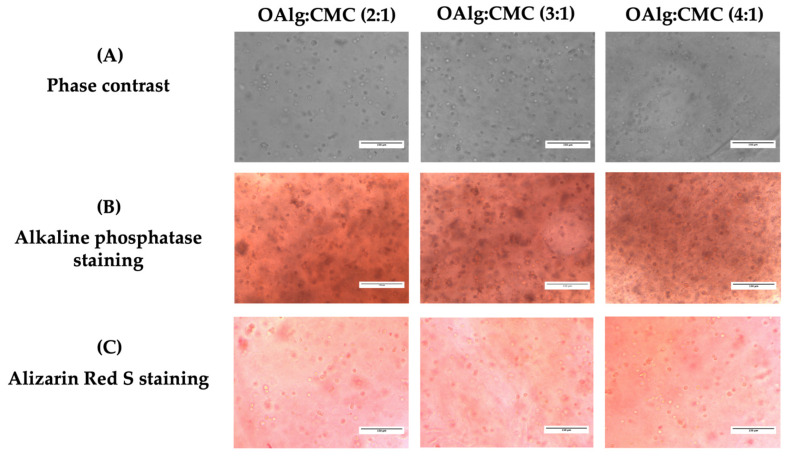
In vitro culture of the dental epithelial cell line HAT-7 in self-crosslinked oxidized alginate-carboxymethyl chitosan hydrogels with three weight ratios of 2:1, 3:1, and 4:1 for 14 days. (**A**) Observation of cell morphology in cell-laden hydrogels using optical phase contrast microscopy. (**B**) Alkaline phosphatase and (**C**) Alizarin red S staining (markers of maturation stage ameloblasts) of HAT-7 cells in self-crosslinked hydrogels, showed initiation of mineralization and ameloblast differentiation.

After 14 days of culture, the capacity of HAT-7 cells cultured in self-crosslinked hydrogels to differentiate into enamel-producing ameloblasts and deposit calcium was assessed by alkaline phosphatase (ALP) and alizarin red S staining, respectively. Cells encapsulated in self-crosslinkable hydrogels with all three different OAlg-CMC weight ratios stained positive with both ALP and alizarin red S (Figure 9B,C). However, no significant difference was observed between the three groups of hydrogels.

### 3.11. SEM Analysis of Cell-Laden Hydrogels

**Figure 10:**
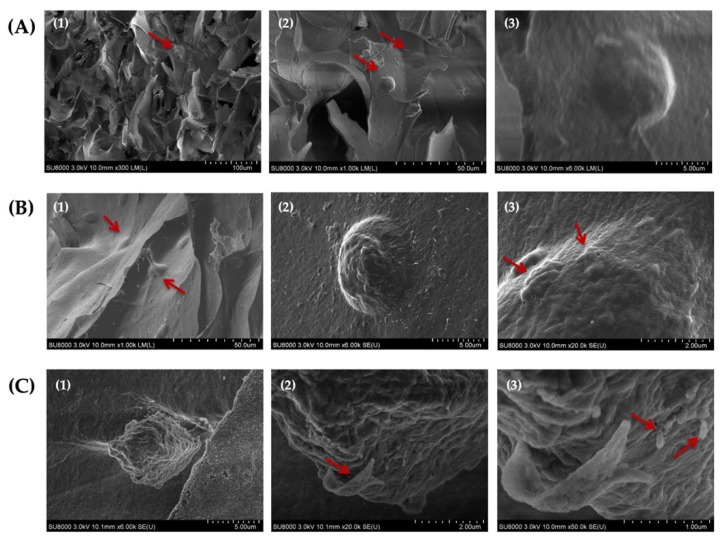
Scanning electron microscopy (SEM) images of HAT-7 cells encapsulated in self-crosslinkable oxidized alginate-carboxymethyl chitosan hydrogel with weight ratio of 2:1(**A1**–**A3**), 3:1 (**B1**–**B3**), and 4:1 (**C1**–**C3**) after 14 days of in vitro culture showed the round morphology of HAT-7 cells and initiation of the mineral formation. (2) and (3) are the magnified (with 10 and 50 k magnifications, respectively) images of (1) and (2). Red arrows show HAT-7 cells in the hydrogels in (**A1**,**A2**,**B1**) and globular-like structures in (**B3**) and ribbon-like and rod-like mineralized structures in (**C2**,**C3**).

The assessment of the morphology of HAT-7 cells cultured in self-crosslinked hydrogels for 14 days using SEM analysis indicated that the cells maintained their round morphology during this entire time period (Figure 10A–C). In addition, our analysis revealed the appearance of globular- and ribbon-like structures and rod-like crystals on the surface of HAT-7 cells (Figure 10(B3,C2,C3)). These mineralized structures suggested that HAT-7 cells started to differentiate into ameloblasts in vitro.

## 4. Discussion

The present study aimed to develop in situ-forming hydrogels composed of oxidized alginate and carboxymethyl chitosan and investigate their suitability for the use as carriers for dental epithelial cells with stem cell characteristics to promote in vitro enamel tissue regeneration. Although self-crosslinkable oxidized alginate-carboxymethyl chitosan hydrogels were previously developed for different applications including wound healing, drug delivery, and bone tissue engineering [23,61,62,63,64], to the best of our knowledge this is the first study on its application for in vitro dental tissue regeneration.

The first step was to modify alginate composition through meta-periodate oxidation to have the aldehyde functional groups required for Schiff base crosslinking. The periodate as the oxidant cleaves the vicinal glycols in the alginate backbone to form dialdehyde groups and generate oxidized alginate; one molecule of sodium meta-periodate was reported to be consumed per α-glycol group [65]. Several factors have been reported to affect the oxidation reaction that include the meta-periodate/alginate ratio, the alginate concentration, the reaction medium, the reaction time, etc. [54,66]. An increase in the meta-periodate/alginate ratio and the alginate concentration were found to increase the degree of oxidation [48,54]. The aqueous solutions of alginate, even at low concentrations, have a very high viscosity and produce a small quantity of oxidized alginate, whereas alginate dispersion in the ethanol–water reaction medium was found to be able to produce a large quantity of oxidized alginate with a higher reaction yield and a lower volume of the solvent [45,66]. In addition, the oxidized alginate synthesized in the ethanol–water mixture showed a higher crosslinking efficiency for hydrogel formation with amino groups containing polymers [66]. The degree of oxidation was reported to increase as the time of the oxidation reaction increased and that the increase in the degree of oxidation is fast in the beginning of the reaction and then slows down in both water and ethanol/water as the reaction mixture in which the reactions takes place [46,66]. According to these studies, a 6-h reaction time is sufficient for a high yield of oxidation reactions. In our study, sodium alginate was oxidized by sodium meta-periodate (with a molar ratio of 0.6 sodium meta-periodate to repeating units of alginate) in an ethanol–water (1:1) mixture for 6 h.

Our Fourier Transform Infrared (FTIR) analysis revealed that the characteristic peaks of alginate at 1605 and 1405 cm^−1^ are still present in the spectrum of oxidized alginate that according to previous studies indicated that the oxidation reaction has no influence on the carboxyl groups in the backbone of alginate [54]. The narrower OH stretching peak that was observed in the spectrum of oxidized alginate in our study showed the contribution of the hydroxyl group in the oxidation reaction, which is in agreement with other studies [46]. The reduced symmetrical C-O-C stretching in the spectrum of oxidized alginate compared to alginate in our study showed the cleavage of the alginate chains as a result of the oxidation reaction according to previous studies [54]. The absence of a new peak at 1735 cm^−1^ in the FTIR spectrum of oxidized alginate that is known to be ascribed to aldehyde groups could be due to the formation of a hemiacetal, which is known as a complex formed between free aldehyde groups of oxidized alginate and the hydroxyl groups of neighboring unoxidized alginate, which hinders the completion of oxidation as previously reported in other studies [54,66,67]. In this study, the appearance of new signals in the ^1^HNMR spectrum of oxidized alginate at 4.2, 5.3, and 5.6 ppm further confirmed the modification of alginate with aldehyde groups, as previously reported in other studies [55]. The oxidation degree is defined as the number of C2–C3 bonds that are cleaved and converted to aldehyde groups [68].

Two major approaches have been previously used in other studies to determine the oxidation degree of alginate [69]. One of these standardized approaches that we used in this study to determine the degree of oxidation of alginate was based on previously published studies by Gomez et al. [48] and consisted of measuring the consumption of the sodium meta-periodate in the oxidation reaction. The experimental oxidation degree was 59.33%, which is in the similar range reported by previous studies [32,48]. We then prepared the hydrogels by mixing the aqueous solutions of oxidized alginate and carboxymethyl chitosan followed by incubation at 37 °C. The kinetics of crosslinking through the Schiff base reaction were previously found by other studies to be contingent upon the pH, temperature, and the type and content of amine and aldehyde groups in the polymers [41,70]. 

The properties of the hydrogel depends on the oxidation degree, and the ratio of aldehyde- to amino-containing polymers [41,45,71]. Thus, in this study, we aimed to modulate the properties of the hydrogels including the gelation time and swelling ability by varying the OAlg:CMC ratios. The chemical composition of self-crosslinked hydrogels with the OAlg:CMC ratio of 4:1, which was expected to have a higher crosslinking density and thus show a stronger peak related to Schiff base linkages, was further analyzed using FTIR. In our study, the appearance of a new peak at 1621 cm^−1^ that is known to represent the imine bonds, indicated the successful Schiff base crosslinking [56]. The observation of the peaks corresponded to aldehyde groups at 1729 cm^−1^ in the spectrum of the hydrogel, and not in the spectrum of oxidized alginate, could be due to the difference in the moisture content of the samples, as previously reported in other studies [69].

Porosity, pore size, and interconnectivity are the important morphological characteristics of tissue engineering scaffolds that influence the migration and proliferation of cells as well as tissue formation [72,73]. Our SEM analysis of hydrogels’ morphology showed the highly porous structures of hydrogels, which indicates the hydrogels’ ability to support tissue reconstitution. The high pore interconnectivity of the hydrogels that was observed in the SEM images can also suggest a high propensity for nutrients exchange [24]. In addition, the pore size of our hydrogels were in the normal range size (at micron scale) of most mammalian cells [74], which suggests the suitability of these hydrogels for promoting and supporting cell proliferation. The wider range of porosity distribution and smaller pore size observed in our hydrogels with a higher concentration of OAlg could be attributed to a faster gelation time and a higher crosslinking density.

The injectability test of the hydrogels showed their potential to be used to encapsulate cells homogenously and to be directly injected to the defect site [51]. The self-healing assays of the hydrogels confirmed the spontaneous ability of the OAlg-CMC hydrogels to repair themselves quickly once they are fractured under external forces, which help them to maintain their structural stability and mechanical strength [51].

The zone inhibition test revealed the antibacterial activity of the OAlg-CMC hydrogels at all three different mixture ratios towards *Streptococcus mutans* and *Streptococcus sobrinus*, known as the main bacterial strains that contribute to enamel demineralization [75]. The antibacterial activity of the hydrogels could be attributed to the presence of chitosan and the Schiff base reaction. The previous studies have reported the antibacterial and antibiofilm activities against cariogenic microorganisms including *Streptococcus mutans* and *Streptococcus sobrinus* [76,77]. The antibacterial effect of chitosan is due to the electrostatic interactions between the positive charges of amino groups in chitosan and the negatively charged bacterial walls [78]. In addition, Schiff base reactions have been found to significantly increase the antimicrobial effects of chitosan [52,79].

Our live/dead assay revealed that self-crosslinkable hydrogels provide a favorable 3D environment for cell survival in the absence of cytotoxic effects. No decrease in the viability of cells within the injected hydrogels was observed compared to hydrogels that were formed by mixing the hydrogel components in the wells without injection. This finding is in agreement with other studies reporting no cell damage after cells have been extruded in the self-healing injectable carboxyethyl chitosan and oxidized alginate from a syringe through the 21-gauge needle [26]. It was previously reported that although oxidized alginate has a slightly higher cytotoxic effect compared to alginate, the crosslinking of OAlg with CMC decreased the cellular toxicity due to the consumption of free aldehyde groups in OAlg in the crosslinking reactions [62].

Alkaline phosphatase, a marker of enamel matrix mineralization [80], was found to be expressed in all groups of cell-laden hydrogels with three different ratios of OAlg:CMC after 14 days of culture, indicating hydrogels’ ability in supporting differentiation and mineralization of dental epithelial stem cells. The HAT-7 cells encapsulated in all groups of the self-crosslinkable hydrogels were stained positively with alizarin red S, which detects calcium deposition [81]. However, no difference in the intensity of ALP and alizarin red S staining were noted between the three groups of hydrogels, which might be due to the absence of differentiation-inducing factors in the culture media.

Remarkably, SEM images of encapsulated HAT-7 cells in self-crosslinked hydrogels showed the ability of these cells to retain their normal round morphology inside the hydrogels after 14 days of in vitro culture. This is consistent with another study that also reported the round morphology of HAT-7 cells in chitosan-collagen I hydrogels [82]. In addition, the formation of rod-and ribbon-like structures on the cell surface observed in the SEM images further confirmed the initiation of mineralization and differentiation in vitro of dental epithelial stem cells, which is in agreement with the ALP and alizarin red staining results.

Taken together, our results of SEM images, ALP, and alizarin red staining demonstrated the ability of self-crosslinked hydrogels to support in vitro mineralization and enamel-like tissue formation. Notably, this study lays a foundation for injection cell therapy for the regeneration of dental enamel to potentially treat dental defects. However, there are some limitations associated to this study, which could be addressed by future research. These limitations include the lack of assessment of the expression of ameloblast markers in mRNA and protein levels, and the difference between the development period in rat-derived dental epithelial HAT-7 cells compared to human epithelial cells. More studies are urged to further investigate the potential for differentiation and mineralization of HAT-7 cells cultured in self-crosslinkable hydrogels, in parallel with ex vivo and in vivo studies to evaluate the suitability of the in situ-forming hydrogels for clinical applications. Furthermore, the study of the hydrogels’ stiffness for dental defects will be an interesting research topic. With the advances from these future studies, the key idea of the present paper, i.e., encapsulating dental epithelial cells in the oxidized alginate-CMC hydrogel, holds promises for translation in clinical therapeutic applications applied to restorative dentistry.

## 5. Conclusions

In this work, injectable self-healing hydrogels were prepared by Schiff base self-crosslinking reactions between aldehyde groups in oxidized alginate and amino groups in carboxymethyl chitosan and characterized for their suitability for enamel dental tissue engineering applications. The hydrogels’ properties were fine-tuned by changing the oxidized alginate to carboxymethyl chitosan weight ratio from 4:1, 3:1, to 2:1 and the resulting hydrogels were quickly (less than 5 min) formed making them more suitable for clinical applications. The hydrogels’ gelation time and swelling degree decreased as the weight ratio of oxidized alginate to carboxymethyl chitosan increased. The hydrogels’ highly porous structure enhanced their ability to support cell migration and proliferation and to facilitate nutrients and fluids exchange. The self-crosslinkable hydrogels showed a rapid self-healing ability after injection due to their dynamic imine bonds. The hydrogels’ antibacterial activity against cariogenic bacteria reinforced their potential as a cell carrier system while decreasing the risk of bacterial infection during tissue regeneration. The sustained high in vitro viability of the dental epithelial stem cell line HAT-7 cells, when encapsulated and injected within the self-crosslinkable hydrogels noted after 14 days of culture, supports the hydrogels suitability as a cell delivery vehicle and injection cell therapy. In addition, the self-crosslinkable hydrogels were able to support HAT-7 cell survival and differentiation potential, indicated by their ability to maintain a round morphology and deposit minerals.

## Figures and Tables

**Table 1:** Labels for different hydrogel groups based on their compositions.

Label	Weight Ratio		Final Concentration (*w*/*v*%)		Total Polymer Concentration (*w*/*v*%)
	OAlg	CMC	OAlg	CMC	
OAlg:CMC (4:1)	4	1	12	3	15
OAlg:CMC (3:1)	3	1	9	3	12
OAlg:CMC (2:1)	2	1	6	3	9

## Data Availability

The data that support the findings of this study are available upon reasonable request from corresponding author.

## References

[1] Kim D., Barraza J.P., Arthur R.A., Hara A., Lewis K., Liu Y., Scisci E.L., Hajishengallis E., Whiteley M., Koo H. (2020). Spatial mapping of polymicrobial communities reveals a precise biogeography associated with human dental caries. Proc. Natl. Acad. Sci. USA.

[2] Qiu W., Zhou Y., Li Z., Huang T., Xiao Y., Cheng L., Peng X., Zhang L., Ren B. (2020). Application of antibiotics/antimicrobial agents on dental caries. Biomed Res. Int..

[3] Binder M., Biggs L.C., Kronenberg M.S., Schneider P., Thesleff I., Balic A. (2020). Novel strategies for expansion of tooth epithelial stem cells and ameloblast generation. Sci. Rep..

[4] Honda M.J., Shinmura Y., Shinohara Y. (2009). Enamel tissue engineering using subcultured enamel organ epithelial cells in combination with dental pulp cells. Cells Tissues Organs.

[5] Yazdanian M., Arefi A.H., Alam M., Abbasi K., Tebyaniyan H., Tahmasebi E., Ranjbar R., Seifalian A., Yazdanian A. (2021). Decellularized and biological scaffolds in dental and craniofacial tissue engineering: A comprehensive overview. J. Mater. Res. Technol..

[6] Chatzistavrou X., Papagerakis S., Ma P.X., Papagerakis P. (2012). Innovative approaches to regenerate enamel and dentin. Int. J. Dent..

[7] Rosa V., Della Bona A., Cavalcanti B.N., Nör J.E. (2012). Tissue engineering: From research to dental clinics. Dent. Mater..

[8] Dong Q., Wang Y., Mohabatpour F., Zheng L., Papagerakis S., Chen D., Papagerakis P. (2019). Dental Pulp Stem Cells: Isolation, Characterization, Expansion, and Odontoblast Differentiation for Tissue Engineering. Odontogenesis.

[9] Fang Z., Guo M., Zhou Q., Li Q., Wong H.M., Cao C.Y. (2021). Enamel-like tissue regeneration by using biomimetic enamel matrix proteins. Int. J. Biol. Macromol..

[10] Duailibi M.T., Duailibi S.E., Dantas F.M.L., Yelick P.C. (2019). Scaffolds that promote enamel remineralization. Handbook of Tissue Engineering Scaffolds: Volume One.

[11] Mukherjee K., Ruan Q., Liberman D., White S.N., Moradian-Oldak J. (2016). Repairing human tooth enamel with leucine-rich amelogenin peptide–chitosan hydrogel. J. Mater. Res..

[12] Fang R., Tian W., Chen X. (2017). Synthesis of injectable alginate hydrogels with muscle-derived stem cells for potential myocardial infarction repair. Appl. Sci..

[13] Qiao S., Zhao Y., Li C., Yin Y., Meng Q., Lin F.-H., Liu Y., Hou X., Guo K., Chen X. (2016). An alginate-based platform for cancer stem cell research. Acta Biomater..

[14] Fang R., Qiao S., Liu Y., Meng Q., Chen X., Song B., Hou X., Tian W. (2015). Sustained co-delivery of BIO and IGF-1 by a novel hybrid hydrogel system to stimulate endogenous cardiac repair in myocardial infarcted rat hearts. Int. J. Nanomed..

[15] Bai X., Fang R., Zhang S., Shi X., Wang Z., Chen X., Yang J., Hou X., Nie Y., Li Y. (2013). Self-cross-linkable hydrogels composed of partially oxidized alginate and gelatin for myocardial infarction repair. J. Bioact. Compat. Polym..

[16] Chang B., Ahuja N., Ma C., Liu X. (2017). Injectable scaffolds: Preparation and application in dental and craniofacial regeneration. Mater. Sci. Eng. R Rep..

[17] Toh W. (2014). Injectable hydrogels in dentistry: Advances and promises. Austin J. Dent..

[18] Haugen H.J., Basu P., Sukul M., Mano J.F., Reseland J.E. (2020). Injectable Biomaterials for Dental Tissue Regeneration. Int. J. Mol. Sci..

[19] Amini A.A., Nair L.S. (2012). Injectable hydrogels for bone and cartilage repair. Biomed. Mater..

[20] Liu C., Zhang Q., Zhu S., Liu H., Chen J. (2019). Preparation and applications of peptide-based injectable hydrogels. RSC Adv..

[21] Nguyen Q.V., Park J.H., Lee D.S. (2015). Injectable polymeric hydrogels for the delivery of therapeutic agents: A review. Eur. Polym. J..

[22] Mathew A.P., Uthaman S., Cho K.-H., Cho C.-S., Park I.-K. (2018). Injectable hydrogels for delivering biotherapeutic molecules. Int. J. Biol. Macromol..

[23] Ma L., Su W., Ran Y., Ma X., Yi Z., Chen G., Chen X., Deng Z., Tong Q., Wang X. (2020). Synthesis and characterization of injectable self-healing hydrogels based on oxidized alginate-hybrid-hydroxyapatite nanoparticles and carboxymethyl chitosan. Int. J. Biol. Macromol..

[24] Naghizadeh Z., Karkhaneh A., Khojasteh A. (2018). Self-crosslinking effect of chitosan and gelatin on alginate based hydrogels: Injectable in situ forming scaffolds. Mater. Sci. Eng. C.

[25] Talebian S., Mehrali M., Taebnia N., Pennisi C.P., Kadumudi F.B., Foroughi J., Hasany M., Nikkhah M., Akbari M., Orive G. (2019). Self-healing hydrogels: The next paradigm shift in tissue engineering?. Adv. Sci..

[26] Wei Z., Zhao J., Chen Y.M., Zhang P., Zhang Q. (2016). Self-healing polysaccharide-based hydrogels as injectable carriers for neural stem cells. Sci. Rep..

[27] Pathan N., Shende P. (2021). Strategic conceptualization and potential of self-healing polymers in biomedical field. Mater. Sci. Eng. C.

[28] Ding F., Wu S., Wang S., Xiong Y., Li Y., Li B., Deng H., Du Y., Xiao L., Shi X. (2015). A dynamic and self-crosslinked polysaccharide hydrogel with autonomous self-healing ability. Soft Matter.

[29] Fan L., Pan X., Zhou Y., Chen L., Xie W., Long Z., Zheng H. (2011). Preparation and characterization of crosslinked carboxymethyl chitosan–oxidized sodium alginate hydrogels. J. Appl. Polym. Sci..

[30] Naghieh S., Sarker M.D., Sharma N.K., Barhoumi Z., Chen X. (2020). Printability of 3D printed hydrogel scaffolds: Influence of hydrogel composition and printing parameters. Appl. Sci..

[31] Chen D.X.B. (2019). Extrusion Bioprinting of Scaffolds for Tissue Engineering Applications.

[32] Baniasadi H., Mashayekhan S., Fadaoddini S., Haghirsharifzamini Y. (2016). Design, fabrication and characterization of oxidized alginate–gelatin hydrogels for muscle tissue engineering applications. J. Biomater. Appl..

[33] Soltan N., Ning L., Mohabatpour F., Papagerakis P., Chen X. (2019). Printability and cell viability in bioprinting alginate dialdehyde-gelatin scaffolds. ACS Biomater. Sci. Eng..

[34] Shariatinia Z. (2018). Carboxymethyl chitosan: Properties and biomedical applications. Int. J. Biol. Macromol..

[35] Sadeghianmaryan A., Naghieh S., Yazdanpanah Z., Sardroud H.A., Sharma N.K., Wilson L.D., Chen X. (2022). Fabrication of chitosan/alginate/hydroxyapatite hybrid scaffolds using 3D printing and impregnating techniques for potential cartilage regeneration. Int. J. Biol. Macromol..

[36] Sadeghianmaryan A., Naghieh S., Sardroud H.A., Yazdanpanah Z., Soltani Y.A., Sernaglia J., Chen X. (2020). Extrusion-based printing of chitosan scaffolds and their in vitro characterization for cartilage tissue engineering. Int. J. Biol. Macromol..

[37] Bakhsheshi-Rad H.R., Ismail A.F., Aziz M., Akbari M., Hadisi Z., Omidi M., Chen X. (2020). Development of the PVA/CS nanofibers containing silk protein sericin as a wound dressing: In vitro and in vivo assessment. Int. J. Biol. Macromol..

[38] Palma P.J., Ramos J.C., Martins J.B., Diogenes A., Figueiredo M.H., Ferreira P., Viegas C., Santos J.M. (2017). Histologic evaluation of regenerative endodontic procedures with the use of chitosan scaffolds in immature dog teeth with apical periodontitis. J. Endod..

[39] Pandit A.H., Mazumdar N., Imtiyaz K., Alam Rizvi M.M., Ahmad S. (2020). Self-healing and injectable hydrogels for anticancer drug delivery: A study with multialdehyde gum arabic and succinic anhydride chitosan. ACS Appl. Bio Mater..

[40] Xu H., Zhang L., Cai J. (2018). Injectable, self-healing, β-chitin-based hydrogels with excellent cytocompatibility, antibacterial activity, and potential as drug/cell carriers. ACS Appl. Bio Mater..

[41] Luü S., Gao C., Xu X., Bai X., Duan H., Gao N., Feng C., Xiong Y., Liu M. (2015). Injectable and self-healing carbohydrate-based hydrogel for cell encapsulation. ACS Appl. Mater. Interfaces.

[42] Li Y., Zhang Y., Shi F., Tao L., Wei Y., Wang X. (2017). Modulus-regulated 3D-cell proliferation in an injectable self-healing hydrogel. Colloids Surf. B Biointerfaces.

[43] Qu J., Zhao X., Liang Y., Zhang T., Ma P.X., Guo B. (2018). Antibacterial adhesive injectable hydrogels with rapid self-healing, extensibility and compressibility as wound dressing for joints skin wound healing. Biomaterials.

[44] Wang K., Dong R., Tang J., Li H., Dang J., Zhang Z., Yu Z., Guo B., Yi C. (2022). Exosomes laden self-healing injectable hydrogel enhances diabetic wound healing via regulating macrophage polarization to accelerate angiogenesis. Chem. Eng. J..

[45] Sarker B., Papageorgiou D.G., Silva R., Zehnder T., Gul-E-Noor F., Bertmer M., Kaschta J., Chrissafis K., Detsch R., Boccaccini A.R. (2014). Fabrication of alginate–gelatin crosslinked hydrogel microcapsules and evaluation of the microstructure and physico-chemical properties. J. Mater. Chem. B.

[46] You F., Wu X., Kelly M., Chen X. (2020). Bioprinting and in vitro characterization of alginate dialdehyde–gelatin hydrogel bio-ink. Bio-Des. Manuf..

[47] Peer M.S., Kasimani R., Rajamohan S., Ramakrishnan P. (2017). Experimental evaluation on oxidation stability of biodiesel/diesel blends with alcohol addition by rancimat instrument and FTIR spectroscopy. J. Mech. Sci. Technol..

[48] Gomez C.G., Rinaudo M., Villar M.A. (2007). Oxidation of sodium alginate and characterization of the oxidized derivatives. Carbohydr. Polym..

[49] Mousavi A., Mashayekhan S., Baheiraei N., Pourjavadi A. (2021). Biohybrid oxidized alginate/myocardial extracellular matrix injectable hydrogels with improved electromechanical properties for cardiac tissue engineering. Int. J. Biol. Macromol..

[50] Huang W., Wang Y., Huang Z., Wang X., Chen L., Zhang Y., Zhang L. (2018). On-demand dissolvable self-healing hydrogel based on carboxymethyl chitosan and cellulose nanocrystal for deep partial thickness burn wound healing. ACS Appl. Mater. Interfaces.

[51] Huang W., Wang Y., Chen Y., Zhao Y., Zhang Q., Zheng X., Chen L., Zhang L. (2016). Strong and rapidly self-healing hydrogels: Potential hemostatic materials. Adv. Healthc. Mater..

[52] Li H., Cheng F., Wei X., Yi X., Tang S., Wang Z., Zhang Y.S., He J., Huang Y. (2021). Injectable, self-healing, antibacterial, and hemostatic N, O-carboxymethyl chitosan/oxidized chondroitin sulfate composite hydrogel for wound dressing. Mater. Sci. Eng. C.

[53] Kawano S., Morotomi T., Toyono T., Nakamura N., Uchida T., Ohishi M., Toyoshima K., Harada H. (2002). Establishment of Dental Epithelial Cell Line (HAT-7) and the Cell Differentiation Dependent on Notch Signaling Pathway. Connect. Tissue Res..

[54] Emami Z., Ehsani M., Zandi M., Foudazi R. (2018). Controlling alginate oxidation conditions for making alginate-gelatin hydrogels. Carbohydr. Polym..

[55] Park J., Nam J., Yun H., Jin H.-J., Kwak H.W. (2021). Aquatic polymer-based edible films of fish gelatin crosslinked with alginate dialdehyde having enhanced physicochemical properties. Carbohydr. Polym..

[56] Wu Y., Yuan L., Sheng N., Gu Z., Feng W., Yin H., Morsi Y., Mo X. (2017). A soft tissue adhesive based on aldehyde-sodium alginate and amino-carboxymethyl chitosan preparation through the Schiff reaction. Front. Mater. Sci..

[57] Kurniasih M., Cahyati T., Dewi R.S. (2018). Carboxymethyl chitosan as an antifungal agent on gauze. Int. J. Biol. Macromol..

[58] Nguyen T.-P., Lee B.-T. (2012). Fabrication of oxidized alginate-gelatin-BCP hydrogels and evaluation of the microstructure, material properties and biocompatibility for bone tissue regeneration. J. Biomater. Appl..

[59] Liao H., Zhang H., Chen W. (2009). Differential physical, rheological, and biological properties of rapid in situ gelable hydrogels composed of oxidized alginate and gelatin derived from marine or porcine sources. J. Mater. Sci. Mater. Med..

[60] Conrads G., de Soet J.J., Song L., Henne K., Sztajer H., Wagner-Döbler I., Zeng A.-P. (2014). Comparing the cariogenic species Streptococcus sobrinus and S. mutans on whole genome level. J. Oral Microbiol..

[61] Chen H., Xing X., Tan H., Jia Y., Zhou T., Chen Y., Ling Z., Hu X. (2017). Covalently antibacterial alginate-chitosan hydrogel dressing integrated gelatin microspheres containing tetracycline hydrochloride for wound healing. Mater. Sci. Eng. C.

[62] Li X., Weng Y., Kong X., Zhang B., Li M., Diao K., Zhang Z., Wang X., Chen H. (2012). A covalently crosslinked polysaccharide hydrogel for potential applications in drug delivery and tissue engineering. J. Mater. Sci. Mater. Med..

[63] Ren B., Chen X., Du S., Ma Y., Chen H., Yuan G., Li J., Xiong D., Tan H., Ling Z. (2018). Injectable polysaccharide hydrogel embedded with hydroxyapatite and calcium carbonate for drug delivery and bone tissue engineering. Int. J. Biol. Macromol..

[64] Li X., Chen S., Zhang B., Li M., Diao K., Zhang Z., Li J., Xu Y., Wang X., Chen H. (2012). In situ injectable nano-composite hydrogel composed of curcumin, N, O-carboxymethyl chitosan and oxidized alginate for wound healing application. Int. J. Pharm..

[65] Balakrishnan B., Jayakrishnan A. (2005). Self-cross-linking biopolymers as injectable in situ forming biodegradable scaffolds. Biomaterials.

[66] Balakrishnan B., Lesieur S., Labarre D., Jayakrishnan A. (2005). Periodate oxidation of sodium alginate in water and in ethanol–water mixture: A comparative study. Carbohydr. Res..

[67] Rajalekshmi R., Shaji A.K., Joseph R., Bhatt A. (2021). Scaffold for liver tissue engineering: Exploring the potential of fibrin incorporated alginate dialdehyde–gelatin hydrogel. Int. J. Biol. Macromol..

[68] Höglund E. (2015). Production of Dialdehyde Cellulose and Periodate Regeneration: Towards Feasible Oxidation Processes. Ph.D. Thesis.

[69] Reakasame S., Boccaccini A.R. (2018). Oxidized alginate-based hydrogels for tissue engineering applications: A review. Biomacromolecules.

[70] Astudillo-Ortiz E., Babo P.S., Reis R.L., Gomes M.E. (2021). Evaluation of Injectable Hyaluronic Acid-Based Hydrogels for Endodontic Tissue Regeneration. Materials.

[71] Liu J., Li J., Yu F., Zhao Y., Mo X., Pan J. (2020). In situ forming hydrogel of natural polysaccharides through Schiff base reaction for soft tissue adhesive and hemostasis. Int. J. Biol. Macromol..

[72] Sarker B., Li W., Zheng K., Detsch R., Boccaccini A.R. (2016). Designing porous bone tissue engineering scaffolds with enhanced mechanical properties from composite hydrogels composed of modified alginate, gelatin, and bioactive glass. ACS Biomater. Sci. Eng..

[73] Kim K., Yeatts A., Dean D., Fisher J.P. (2010). Stereolithographic bone scaffold design parameters: Osteogenic differentiation and signal expression. Tissue Eng. Part B Rev..

[74] Zhang L., Liu J., Zheng X., Zhang A., Zhang X., Tang K. (2019). Pullulan dialdehyde crosslinked gelatin hydrogels with high strength for biomedical applications. Carbohydr. Polym..

[75] Ahn S.-J., Lim B.-S., Lee S.-J. (2007). Prevalence of cariogenic streptococci on incisor brackets detected by polymerase chain reaction. Am. J. Orthod. Dentofac. Orthop..

[76] Abedian Z., Jenabian N., Moghadamnia A.A., Zabihi E., Tashakorian H., Rajabnia M., Sadighian F., Bijani A. (2019). Antibacterial activity of high-molecular-weight and low-molecular-weight chitosan upon oral pathogens. J. Conserv. Dent. JCD.

[77] Aliasghari A., Khorasgani M.R., Vaezifar S., Rahimi F., Younesi H., Khoroushi M. (2016). Evaluation of antibacterial efficiency of chitosan and chitosan nanoparticles on cariogenic streptococci: An in vitro study. Iran. J. Microbiol..

[78] Perinelli D.R., Fagioli L., Campana R., Lam J.K.W., Baffone W., Palmieri G.F., Casettari L., Bonacucina G. (2018). Chitosan-based nanosystems and their exploited antimicrobial activity. Eur. J. Pharm. Sci..

[79] Anush S.M., Vishalakshi B., Kalluraya B., Manju N. (2018). Synthesis of pyrazole-based Schiff bases of Chitosan: Evaluation of antimicrobial activity. Int. J. Biol. Macromol..

[80] Yoshizaki K., Hu L., Nguyen T., Sakai K., Ishikawa M., Takahashi I., Fukumoto S., DenBesten P.K., Bikle D.D., Oda Y. (2017). Mediator 1 contributes to enamel mineralization as a coactivator for Notch1 signaling and stimulates transcription of the alkaline phosphatase gene. J. Biol. Chem..

[81] Gregory C.A., Gunn W.G., Peister A., Prockop D.J. (2004). An Alizarin red-based assay of mineralization by adherent cells in culture: Comparison with cetylpyridinium chloride extraction. Anal. Biochem..

[82] Ravindran S., Song Y., George A. (2010). Development of three-dimensional biomimetic scaffold to study epithelial–mesenchymal interactions. Tissue Eng. Part A.

